# Supplementary material for: The causal relationship between the human gut microbiota and pyogenic arthritis: a Mendelian randomization study
Source: Front Cell Infect Microbiol. 2024 Nov 26;14:1452480. doi: 10.3389/fcimb.2024.1452480 (PMC11629706; doi:10.3389/fcimb.2024.1452480)
Supplement: Supplementary file 1 [file DataSheet1.zip › mendelian test/ebi-a-GCST90027608.csv_forest.pdf]

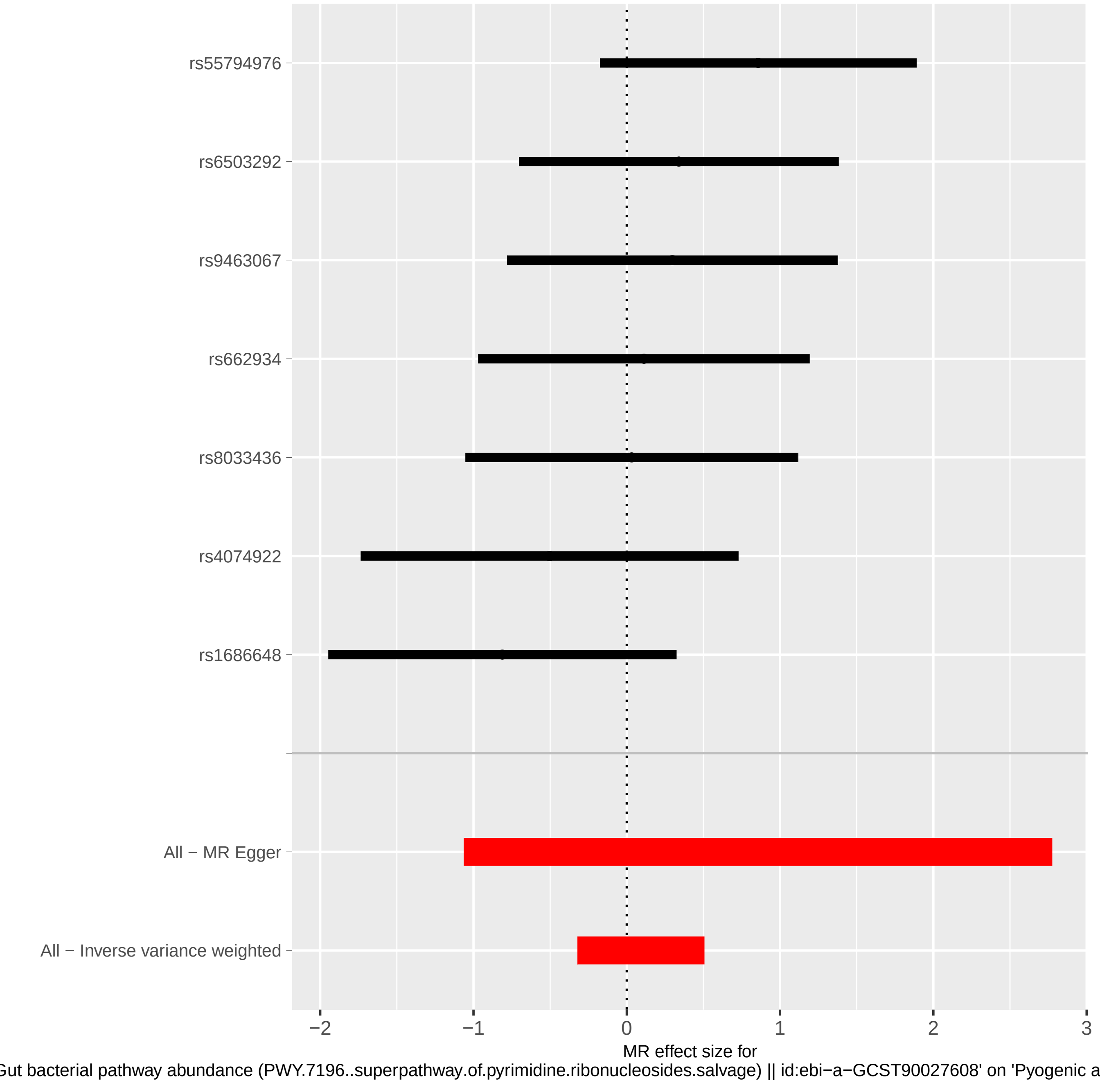
rs55794976
rs6503292
rs9463067
rs662934
rs8033436
rs4074922
rs1686648
All − MR Egger
All − Inverse variance weighted
−2
−1
0
1
2
3
MR effect size for
Gut bacterial pathway abundance (PWY.7196..superpathway.of.pyrimidine.ribonucleosides.salvage) || id:ebi−a−GCST90027608' on 'Pyogenic a
